# Overreporting of adherence to hepatitis C direct-acting antiviral therapy and sustained virologic response among people who inject drugs in the HERO study

**DOI:** 10.1186/s12879-024-09124-3

**Published:** 2024-02-23

**Authors:** Snehal S. Lopes, Irene Pericot-Valverde, Paula J. Lum, Lynn E. Taylor, Shruti H. Mehta, Judith I. Tsui, Judith Feinberg, Arthur Y. Kim, Brianna L. Norton, Kimberly Page, Cristina Murray-Krezan, Jessica Anderson, Alison Karasz, Julia Arnsten, Phillip Moschella, Moonseong Heo, Alain H. Litwin

**Affiliations:** 1 Department of Public Health Sciences, Clemson University, 29634 Clemson, SC USA; 2 Department of Psychology, College of Behavioral, Social, and Health Sciences, Clemson University, 29634 Clemson, SC USA; 3 Department of Medicine, University of California, San Francisco, 1001 Potrero Ave, 94110 San Francisco, CA USA; 4 Department of Pharmacy Practice and Clinical Research, University of Rhode Island, 7 Greenhouse Road, 02881 Kingston, RI USA; 5 Department of Epidemiology, Johns Hopkins Bloomberg School of Public Health, 615 N. Wolfe Street, Room E6546, 21205 Baltimore, MD USA; 6 Department of Medicine, University of Washington, 325 9th Ave, 98104 Seattle, WA USA; 7 Department of Behavioral Medicine and Psychiatry, West Virginia University School of Medicine, 930 Chestnut Ridge Road, 26505 Morgantown, WV USA; 8 Department of Medicine, Section of Infectious Diseases, West Virginia University School of Medicine, 1 Medical Center Drive, 26506 Morgantown, WV USA; 9 Division of Infectious Diseases, Massachusetts General Hospital, 55 Fruit St, 02114 Boston, MA USA; 10 Harvard Medical School, 02115 Boston, MA USA; 11 Albert Einstein College of Medicine, 10461 Bronx, NY USA; 12 Department of Medicine, Montefiore Medical Center, 10467 Bronx, NY USA; 13 Department of Internal Medicine, Health Sciences Center, University of New Mexico, University of New Mexico, MSC 10, 5550, 87131 Albuquerque, NM USA; 14 Division of General Internal Medicine, Department of Medicine, University of Pittsburgh School of Medicine, 15213 Pittsburgh, PA USA; 15 UMass Chan Medical School, University of Massachusetts Medical School, 55 Lake Ave, North, 01605 Worcester, MA USA; 16 Department of Emergency Medicine, Prisma Health, Greenville, SC USA; 17 School of Health Research, Clemson University, Clemson, SC USA; 18 Department of Medicine, University of South Carolina School of Medicine, 876 W Faris Rd, 29605 Greenville, SC USA; 19 Department of Medicine, Prisma Health, 29605 Greenville, SC USA

**Keywords:** Adherence, Overreporting, Self-report, Objective measure, Visual analog scale, Electronic blister pack, Persons who inject drugs, HCV DAA, SVR

## Abstract

**Background:**

Self-reported adherence to direct-acting antivirals (DAAs) to treat hepatitis C virus (HCV) among persons who inject drugs (PWID) is often an overreport of objectively measured adherence. The association of such overreporting with sustained virologic response (SVR) is understudied. This study among PWID aimed to determine a threshold of overreporting adherence that optimally predicts lower SVR rates, and to explore correlates of the optimal overreporting threshold.

**Methods:**

This study analyzed per-protocol data of participants with adherence data (*N* = 493) from the HERO (Hepatitis C Real Options) study. Self-reported and objective adherence to a 12-week DAA regimen were measured using visual analogue scales and electronic blister packs, respectively. The difference (Δ) between self-reported and objectively measured adherence was calculated. We used the Youden index based on receiver operating characteristic (ROC) curve analysis to identify an optimal threshold of overreporting for predicting lower SVR rates. Factors associated with the optimal threshold of overreporting were identified by comparing baseline characteristics between participants at/above versus those below the threshold.

**Results:**

The self-reported, objective, and Δ adherence averages were 95.1% (SD = 8.9), 75.9% (SD = 16.3), and 19.2% (SD = 15.2), respectively. The ≥ 25% overreporting threshold was determined to be optimal. The SVR rate was lower for ≥ 25% vs. < 25% overreporting (86.7% vs. 95.8%, *p* <.001). The factors associated with ≥ 25% Δ adherence were unemployment; higher number of days and times/day of injecting drugs; higher proportion of positive urine drug screening for amphetamine, methamphetamine, and oxycodone, and negative urine screening for THC (tetrahydrocannabinol)/cannabis.

**Conclusions:**

Self-reported DAA adherence was significantly greater than objectively measured adherence among PWID by 19.2%. Having ≥ 25% overreported adherence was associated with optimal prediction of lower SVR rates. PWID with risk factors for high overreporting may need to be more intensively managed to promote actual adherence.

## Introduction

Persons who inject drugs (PWID) are at a high-risk for hepatitis C virus (HCV) infection [1]. As per the Centers for Disease Control and Prevention (CDC) 2020 surveillance report, the majority of HCV infections in the United States are the consequence of sharing drug injection equipment [2]. The prevalence of HCV antibodies has been estimated at 52.3% among PWID globally [3]. While direct-acting antiviral (DAA) medications for HCV have been found to be highly effective irrespective of current/former substance use status, lower treatment completion rates may affect the cure rates among PWID [4, 5]. Evidence suggests that higher adherence increases the chances of HCV cure among PWID [5], and having at least 50% objectively measured adherence substantially increases the chances of HCV cure among PWID, with a likelihood of achieving cure increasing further for every 5% interval above the 50% objective adherence threshold [6]. Therefore, it is critical to provide PWID with the support needed to ensure optimal adherence [7].

Self-reported adherence measures, such as the visual analog scale (VAS), are easier to implement in clinical settings than objective measures [8]. Previous studies using objective adherence measures such as pill counts among veterans [9] and electronic blister packs among PWID (including active drug users) [10] provide evidence for the validity of the VAS for measuring DAA adherence by showing that self-reported adherence measured using the VAS is associated with objectively measured adherence. However, self-reported measures of adherence have been found to overestimate the actual level of adherence both in the general population [8] and among PWID with recent drug use [11]. The extent of overestimation is an unknown that reduces the utility of the self-report measure. If the level of overreporting of adherence could be estimated and the associated factors identified, PWID receiving DAAs could be supported to attain at least the minimum levels of adherence that optimize the chances of cure.

Prior studies have investigated factors associated with overreporting adherence in other contexts such as antihypertensives [12] and HIV pre-exposure prophylaxis [13, 14], and suggested that factors such as socioeconomic status and health beliefs [12], educational level [13], race [14], and age [13, 14] may predict overreporting of adherence. Exploring factors associated with overreporting could help clinicians look for signs for overreporting adherence and target those individuals for appropriate support to improve adherence and the treatment outcomes. To our knowledge, no studies have explored factors associated with overreporting adherence to DAAs among PWID or other populations living with HCV. Furthermore, the extent to which overreporting is associated with poorer chances of cure or sustained virologic response (SVR) rates has not been examined.

The HERO (Hepatitis C Real Options) study [5] measured adherence through both self-report using VAS and objectively using electronic blister packs. Analyzing these data, the present study aimed to: (1) investigate the extent of discrepancy between self-reported and objective measurements of adherence; (2) determine a threshold of overreporting for optimally predicting a lower SVR rate; and (3) explore the factors associated with the optimal threshold of overreporting.

## Methods

### Study design and sample

This study included a secondary analysis of data from the HERO study (ClinicalTrials.gov, NCT02824640) [5, 15]. The HERO study was a pragmatic randomized clinical trial conducted across eight opioid treatment programs (OTPs) and fifteen community health centers (CHCs) in eight US states among DAA-naïve PWID with active drug injection use within 90 days of screening. Participants were randomized in a 1:1 ratio to two modes of administration: modified Directly Observed Therapy (mDOT) and Patient Navigation (PN). All participants received a 12-week course of sofosbuvir 400 mg and velpatasvir 100 mg fixed-dose combination therapy in electronic blister packs. Participants were given a maximum compensation of $400 ($20 for completing each of 17 research visits, and $5 for returning the electronic blister packs for each of 12 weeks of treatment). Outcomes included and compared the rates of HCV cure, as well as HCV DAA treatment initiation, completion, and adherence between the two study arms.

The analytic sample for this study is based on the per-protocol sample in the HERO study (*N* = 501), defined as those participants who received the treatment condition as assigned to them through the HERO study randomization procedure and had a determined SVR status after the end of treatment [5] and included those who had at least one data point on adherence to DAAs, in the form of both self-report and objective measurement (*N* = 493).

### Measures

#### Participant characteristics

Self-report survey questionnaires were used to collect information on demographic and clinical characteristics and injection drug use. Additionally, data on urine toxicology results for substance use at baseline were extracted from medical chart review. For analysis in this study, we included all available demographic measures: age, race, ethnicity, gender, relationship status, education, housing stability, and employment status. Clinical characteristics included were depression, measured using the 9-item Patient Health Questionnaire (PHQ-9) [16]; anxiety, measured using the 7-item generalized anxiety disorder scale (GAD-7) [17]; and HIV coinfection. Depression has been associated with poorer adherence [18]. Anxiety is correlated with depression, is the most common mental health issue diagnosis along with depression in the HCV-infected population [19, 20], and was associated with poorer adherence in the interferon-era [21]. HIV coinfection may affect adherence due to the pill burden [22]. All substance use behavior measures at baseline were analyzed because substance use behaviors were associated with objective adherence in the parent study [5]. The substance use behavioral assessments included self-reported injection drug use in the past three months (cocaine, heroin, methamphetamine, crack, fentanyl, and polysubstance) and urine drug test positivity (amphetamine, methamphetamine, benzodiazepine, cocaine, THC [tetrahydrocannabinol]/cannabis, opiate, and oxycodone) at baseline.

#### Self-reported DAA adherence

Adherence to the DAA regimen was self-reported at weeks 4, 8, and 12 during the treatment period using a single item VAS instrument: “How much of your pills have you taken in the past 30 days?” The response was a continuous value between 0 and 100%. Overall self-reported adherence was calculated by taking an average of all available data on self-reported adherence. To enable stratified analyses, we created six categorical levels of overall self-reported adherence: <80%, 80–85%, > 85–<90%, 90–95%, > 95–<100%, and 100%.

#### Objective DAA adherence

Adherence was also objectively measured using electronic blister packs that recorded the day and time of medication removal. Our prior studies explored changes in weekly objective adherence over time and found that objectively measured weekly adherence declined over the 12-week treatment period for both arms [5, 23]. In this study, however, we focused on overall objective adherence as it is more relevant for the outcome of SVR than the consistency of week-by-week adherence. Overall objective adherence was calculated by averaging the weekly objective adherence rate for the 12 weeks of treatment.

#### Difference between self-reported and objective adherence

The difference between self-reported and objective adherence was operationalized in the following two ways:

- Delta (Δ) adherence: This variable was a continuous variable calculated as the difference between overall self-reported and overall objective adherence. Positive nonzero values of Δ adherence indicated overreporting, negative values indicated underreporting, and value = 0 indicated correct reporting of HCV DAA adherence.
- Overreporting thresholds: This included six binary variables indicating different overreported adherence thresholds. Six different cutoffs of Δ adherence (≥ 5%, ≥ 10%, ≥ 15%, ≥ 20%, ≥ 25%, and ≥ 30%) were used to create these binary variables.

#### SVR

SVR, defined as having HCV RNA (ribonucleic acid) level below the limit of quantitation (≤ 15 IU/mL), equivalent to cure, was ascertained at least 12 weeks after treatment completion. HCV RNA was tested by Quest Diagnostics using COBAS TaqMan real-time reverse transcriptase polymerase chain reaction assay (Roche Diagnostics, Basel, Switzerland). The window period for SVR determination was set to between 70 and 365 days following HCV treatment completion. SVR was treated as a binary variable in our analyses (no HCV cure = 0 and HCV cure = 1).

### Statistical analyses

Descriptive analyses were conducted using the demographic and clinical characteristics of the sample. The association of continuous self-reported adherence with objective adherence was tested using linear regression. Scatter plots were created to enable visual inspection of the relationship of self-reported adherence with objective adherence and Δ adherence. Δ Adherence was compared between the six categorical levels of self-reported adherence using generalized linear model regression. SVR rates were compared by each of the binary overreporting thresholds, e.g., ≥ 5% vs. < 5%, ≥ 10% vs. < 10%, and so on. To identify an overreporting threshold that is optimally associated with predicting lower SVR, we used the Youden index (= sensitivity + specificity-1) based on receiver operating characteristic (ROC) curve analysis [24]. Using the optimal overreporting threshold determined through the ROC curve analyses, we compared persons falling at/above the threshold vs. those below the threshold with respect to SVR rates, and the baseline demographic and clinical characteristics. For two-sample comparisons, chi-square/Fisher’s exact tests were used for categorical variables, and independent samples t tests/Wilcoxon rank sum tests were used for continuous variables. All analyses were conducted using SAS software, version 9.4 (SAS Institute Inc., Cary, NC).

## Results

### Characteristics of the study sample

Demographic and clinical characteristics of the study sample at baseline are presented in Table 1. The mean (M) age in the study sample was 44.1 [standard deviation (SD) = 11.5] years. The study sample was 72.4% male, 63.6% White, and 22.9% Hispanic. A majority of the sample (87.4%) reported their relationship status as single, separated, divorced, or widowed. The highest level of education completed was high school or less for 61.8% of the sample, 64.8% were unemployed, 57.6% did not have their own transportation, and 48.0% were living in unstable housing conditions.

**Table 1:** Descriptive Characteristics of Study Sample

	Total (*N* = 493; 100%)	< 25% Overreported adherence (*N* = 335; 67.95%)	≥ 25% Overreported adherence (*N* = 158; 32.05%)	p
**Sociodemographic factors:**				
**Age [M (SD)]**	44.1 (11.5)	44.0 (10.9)	44.3 (12.5)	0.761
**Gender**				0.290
*Female*	131 (26.6%)	90 (26.9%)	41 (25.9%)	
*Male*	357 (72.4%)	240 (71.6%)	117 (74.1%)	
*Transgender/Gender Nonconforming*	5 (1.0%)	5 (1.5%)	0 (0.0%)	
**Race**				0.889
*White/Caucasian*	302 (63.6%)	209 (64.3%)	93 (62.0%)	
*Black/African American*	70 (14.7%)	47 (14.5%)	23 (15.3%)	
*Other*	103 (21.7%)	69 (21.2%)	34 (22.7%)	
**Latino/Hispanic Ethnicity**				0.961
*No*	380 (77.1%)	258 (77.0%)	122 (77.2%)	
*Yes*	113 (22.9%)	77 (23.0%)	36 (22.8%)	
**Marital/cohabitation Status**				0.699
*Single/Separated/Divorced/Widowed*	430 (87.4%)	289 (86.5%)	141 (89.2%)	
*Married/living together as married*	57 (11.6%)	41 (12.3%)	16 (10.1%)	
*Other*	5 (1.0%)	4 (1.2%)	1 (0.6%)	
**Education**				0.299
*Less than High school*	117 (23.8%)	81 (24.3%)	36 (22.8%)	
*High school diploma or GED*	187 (38.0%)	133 (39.8%)	54 (34.2%)	
*Some college or more*	188 (38.2%)	120 (35.9%)	68 (43.0%)	
**Living stability** ^**a**^				0.190
*Stable housing*	256 (52.0%)	167 (50.0%)	89 (56.3%)	
*Unstable housing*	236 (48.0%)	167 (50.0%)	69 (43.7%)	
**Availability of transportation**				0.667
*Yes*	207 (42.2%)	136 (40.8%)	71 (44.9%)	
*Maybe, if I can get a ride*	26 (5.3%)	18 (5.4%)	8 (5.1%)	
*Maybe, if public transportation is available*	254 (51.7%)	177 (53.2%)	77 (48.7%)	
*No*	4 (0.8%)	2 (0.6%)	2 (1.3%)	
**Employed with a regular job or informal work**				0.022
*Yes*	173 (35.2%)	129 (38.6%)	44 (28.0%)	
*No*	318 (64.8%)	205 (61.4%)	113 (72.0%)	
**Clinical Characteristics**:				
**Depressive symptoms (PHQ-9) [M (SD)]**	9.9 (6.4)	9.6 (6.3)	10.6 (6.6)	0.170
**Anxiety symptoms (GAD-7) [M (SD)]**	8.4 (6.4)	8.3 (6.0)	8.6 (7.3)	0.941
**HIV coinfection (positive)**				0.706
*No*	286 (80.1%)	196 (80.7%)	90 (78.9%)	
*Yes*	71 (19.9%)	47 (19.3%)	24 (21.1%)	
**Clinical setting type**				0.216
*OTP*	229 (46.5%)	162 (70.7%)	67 (29.3%)	
*CHC*	264 (53.5%)	173 (65.5%)	91 (34.5%)	
**Drug Use Characteristics**:				
**Last drug injection (within 3 months of screening)**				0.032
*0–4 weeks*	366 (74.2%)	237 (70.7%)	129 (81.6%)	
*5–8 weeks*	84 (17.0%)	66 (19.7%)	18 (11.4%)	
*9–12 weeks*	43 (8.7%)	32 (9.6%)	11 (7.0%)	
**Number of days injected drugs in the past 3 months [M (SD)]**	32.3 (30.4)	28.0 (28.7)	41.1 (32.0)	< 0.001
**Times injecting drugs a day [M (SD)]**	2.9 (2.7)	2.7 (2.7)	3.2 (2.6)	0.001
**Substances injected in the past 3 months**				
*Mixture of cocaine and heroin*	122 (26.1%)	80/314 (25.5%)	42/153 (27.5%)	0.649
*Mixture of methamphetamine and heroin*	107 (22.9%)	70/314 (22.3%)	37/153 (24.2%)	0.648
*Heroin*	376 (80.5%)	259/314 (82.5%)	117/153 (76.5%)	0.124
*Methamphetamine*	173 (37.0%)	111/314 (35.4%)	62/153 (40.5%)	0.277
*Cocaine*	136 (29.1%)	89/314 (28.3%)	47/153 (30.7%)	0.596
*Crack*	68 (14.6%)	45/313 (14.4%)	23/153 (15.0%)	0.851
*Fentanyl*	19 (42.2%)	13/34 (38.2%)	6/11 (54.5%)	0.341
*Poly-substances*	275 (58.9%)	182/314 (58.0%)	93/153 (60.8%)	0.561
**Urine drug screen positive results at baseline visit** ^**14**^				
*Any drug*	457 (96.8%)	311/321 (96.9%)	146/151 (96.7%)	0.910
*Amphetamine*	131 (27.8%)	75/321 (23.4%)	56/151 (37.1%)	0.002
*Methamphetamine*	148 (31.4%)	88/321 (27.4%)	60/151 (39.7%)	0.007
*Benzodiazepine*	257 (54.4%)	178/321 (55.5%)	79/151 (52.3%)	0.524
*Cocaine*	195 (41.3%)	131/321 (40.8%)	64/151 (42.4%)	0.746
*THC/Cannabis*	236 (50.0%)	171/321 (53.3%)	65/151 (43.0%)	0.038
*Opiate*	237 (50.2%)	153/321 (47.7%)	84/151 (55.6%)	0.106
*Oxycodone*	127 (26.9%)	75/321 (23.4%)	52/151 (34.4%)	0.011

Notes: Abbreviations [PHQ-9 (Patient Health Questionnaire, 9-item; Kroenke et al., 2001), GAD-7 (General Anxiety Disorder, 7-item; Spitzer et al., 2006), THC (tetrahydrocannabinol), Opioid Treatment Program (OTP), Community Health Center (CHC)]. ^a^Stable housing was defined as having one’s own/rent apartment, room or house, whereas unstable housing was defined as living in a shelter, dormitory/college residence, halfway house, residential treatment facility/program, institution, someone else’s apartment, room or house, on the street/outdoors, other housing type, refusing or not knowing housing information

### Association between Self-reported and objective adherence

The results of our analyses suggested a significant positive association between self-reported and objective adherence. For every 1% increase in self-reported adherence, the objective adherence was higher by an estimated average of 0.7% [95% Confidence Interval (CI): 0.6%, 0.9%; *p* <.001]. The OTP and CHC groups were not significantly different in the average overall self-report [M (SD) = 96% (8%) for OTP vs. 95% (10%) for CHC, *p* =.180) overall objective adherence [M (SD) = 77% (16%) for OTP vs. 75% (16%) for CHC, *p* =.097). In the non-per-protocol sample who initiated treatment, 85 participants had at least one data point available on both self-report and objective measures of adherence. Compared to our study sample, the 85 non-per-protocol participants who initiated treatment had significantly lower overall objective adherence [M (SD) = 67% (20%) vs. 76% (16%), *p* <.001), but not significantly different overall self-report adherence [M (SD) = 94% (14%) vs. 95% (9%) in our study sample, *p* =.553].

### Difference between self-report and objective adherence

**Fig. 1:**
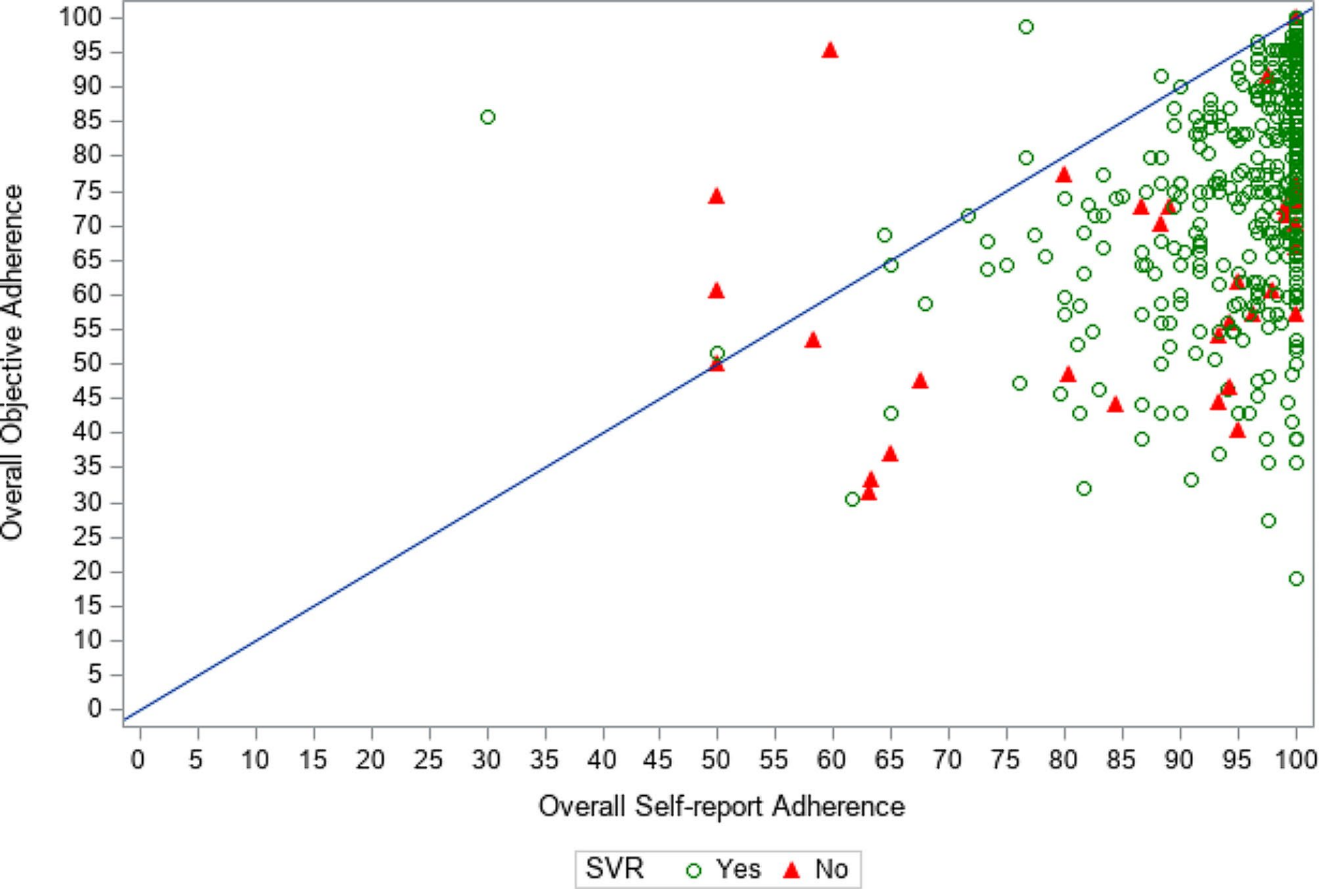
The plot for overall objective adherence by overall self-reported adherence

The scatter plot depicting actual observed values for objective vs. self-reported adherence in Fig. 1 shows that self-reported adherence was overreported by most persons in our study sample compared to their objective adherence values. Table 2 presents the descriptive results for the adherence measures in the total sample and stratified by six categorical levels of self-reported adherence. In the total study sample, the average self-reported adherence was higher in comparison to the average objective adherence [M (SD) = 95.1% (8.9%) vs. 76.0% (16.3%)], and the results were similar within each categorical level of self-reported adherence. The < 80% self-reported adherence category had the smallest average Δ adherence [M (SD) = 5.0% (22.0)]. Compared to the < 80% self-reported adherence category, the average Δ adherence was significantly greater for all of the other self-reported adherence categorical levels.

The markers represent observed values for each of the sample participants. The green and red markers denote participants who did and did not achieve SVR, respectively. The diagonal is the line of equality.

**Table 2:** Self-reported, Objective, and Δ Adherence by Self-reported adherence levels

		**Self-reported Adherence**		**Objective Adherence**		**Δ Adherence** ^**a**^		
**Self-reported levels**	**N**	**M**	**SD**	**M**	**SD**	**M**	**SD**	**p** ^**b**^
**< 80%**	26	65.0	11.8	59.9	18.3	5.0	22.0	(reference)
**80-<85%**	21	82.0	1.5	61.3	13.1	20.7	13.0	0.005
**85-<90%**	27	88.1	1.0	66.1	13.5	21.9	13.2	0.001
**90-<95%**	70	92.8	1.6	68.7	14.7	24.1	14.9	< 0.001
**95-<100%**	146	98.1	1.3	76.9	15.2	21.2	14.9	< 0.001
**100%**	203	100	0	82.7	14.0	17.3	14.0	0.001
**Total**	493	95.1	8.9	76.0	16.3	19.2	15.3	-

Note: ^a^Δ Adherence: The difference between overall self-reported and overall objective adherence;

^b^*p* values for differences in Δ Adherence compared to the < 80% self-report adherence level

### Relation of varying overreporting thresholds with SVR

The results of analyses comparing SVR rates by binary overreporting levels are presented in Table 3. SVR rates were significantly lower for overreporting at ≥ 15% vs. < 15%, ≥ 20% vs. < 20%, ≥ 25% vs. < 25%, and ≥ 30% vs. < 30%. The difference of 9.1% in the SVR rates between the ≥ 25% and < 25% overreporting levels was highly significant (*p* <.001).

**Table 3:** Comparison of SVR rates by varying overreporting thresholds

Δ Adherence^a^	SVR rate: n/N, %		p
< 5%	66/74	89.2%	0.178
≥ 5%	392/419	93.6%	
< 10%	150/159	94.3%	0.391
≥ 10%	308/334	92.2%	
< 15%	216/226	95.6%	0.033
≥ 15%	242/267	90.6%	
< 20%	266/279	95.3%	0.016
≥ 20%	192/214	89.7%	
< 25%	321/335	95.8%	< 0.001
≥ 25%	137/158	86.7%	
< 30%	361/382	94.5%	0.010
≥ 30%	97/111	87.4%	

Note: ^a^Δ Adherence: The difference between overall self-reported and overall objective adherence. Chi-square tests were used for comparisons between groups

### The overreported adherence thresholds for optimally predicting lower SVR rate

Based on the ROC curve analysis, the threshold of ≥ 26.2 Δ adherence had the largest Youden index value of 0.3 for predicting the outcome of SVR. Rounding to the nearest five, a threshold of ≥ 25% Δ adherence was selected as the optimal overreporting threshold to indicate a problematic level of overreporting adherence. In our study sample, 32.1% of the participants had ≥ 25% overreported adherence.

**Fig. 2:**
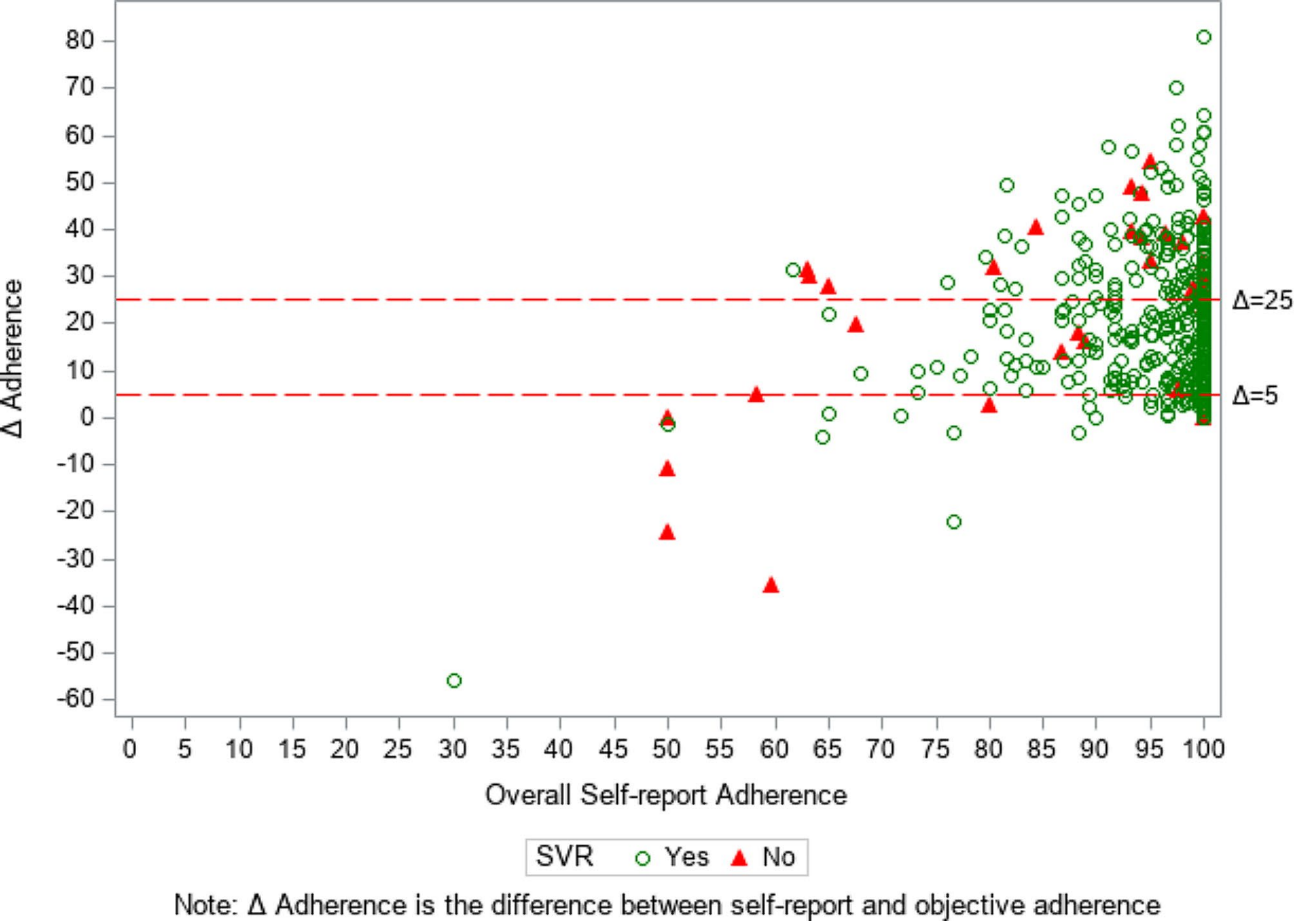
The plot for Δ adherence by overall self-reported adherence

Table 4 shows the differences in SVR rates between ≥ 25% and < 25% overreported adherence groups for the total study sample and stratified by six categorical levels of self-reported adherence. Within the 90‒<95% and 100% self-reported adherence categories, the SVR rate was significantly lower for ≥ 25% vs. < 25% overreported adherence. Figure 2 shows that a majority of those without SVR are positioned above the 25% Δ adherence threshold line whereas a majority of those with SVR are below the 25% Δ adherence threshold line.

**Table 4:** SVR rates by the optimal overreported adherence threshold (25%), stratified by self-report levels

Self-report levels	< 25% Overreported adherence		≥ 25% Overreported adherence		p
	n/N	%	n/N	%	
**< 80%**	14/20	70.0	3/6	50.0	.628^a^
**80-<85%**	13/14	92.9	5/7	71.4	.247^a^
**85-<90%**	15/18	83.3	9/9	100	.529^a^
**90-<95%**	41/41	100	23/29	79.3	.004^a^
**95-<100%**	92/93	98.9	49/53	92.5	.058^a^
**100%**	146/149	98.0	48/54	88.9	.012^a^
**Overall sample**	321/335	95.8	137/158	86.7	<.001^b^

Note: ^a^Fisher’s Exact test; ^b^Chi-square test; column percentages reported

The markers represent observed values for each of the sample participants. The green and red markers denote participants who did and did not achieve SVR, respectively. The 5% and 25% Δ adherence thresholds are shown as red horizontal lines.

### Factors associated with the optimal overreporting threshold (≥ 25%)

A comparison of characteristics by ≥ 25% vs. < 25% overreporting groups is presented in Table 1. Employment status was the only sociodemographic variable associated with ≥ 25% overreporting wherein persons with ≥ 25% overreporting had higher rates of unemployment compared to those below the 25% overreporting level (72.0% vs. 61.4%, *p* =.02).

Various drug-related variables were associated with ≥ 25% overreporting, including the number of days of injecting drugs in the previous 3-months, the number of times per day of injecting drugs, and positive urine test results for drug use. More specifically, the ≥ 25% Δ adherence had a higher average number of days of injecting drugs in the 3-month period before baseline (41.1 vs. 28.0, *p* <.001), a higher average number of times per day of injecting drugs (3.2 vs. 2.7, *p* =.001), a larger proportion of positive urine test results for amphetamine (37.1% vs. 23.4%, *p* =.002), methamphetamine (39.7% vs. 27.4%, *p* =.007), and oxycodone (34.4% vs. 23.4%, *p* =.011), but a smaller proportion of positive urine test results for THC (tetrahydrocannabinol)/cannabis (43.0 vs. 53.3, *p* =.038) compared to those with < 25% Δ adherence.

## Discussion

Although self-reported adherence using the VAS measure was significantly associated with objective adherence measured using electronic blister packs, it overestimated adherence by 19.2% on average. The ≥ 25% overreporting was determined as the optimal threshold for predicting SVR and was associated with lower rates of SVR vs. the < 25% Δ adherence group (86.7% vs. 95.8%). Unemployment, higher rates of substance use behaviors, positive urine drug test results for amphetamine, methamphetamine, and oxycodone, and negative urine drug test results for THC/cannabis predicted having ≥ 25% overreporting of adherence.

There is considerable evidence indicating that self-reported measures for HCV DAA adherence tend to overestimate actual adherence [25]. The findings of our study are consistent with some prior studies that measured objective adherence with electronic blister packs among PWID [10, 26] and studies that measured objective adherence using medication event monitoring system (MEMS) caps and pill counts among the general population treated for chronic HCV [27] where self-reported adherence overestimated actual adherence. Our estimate of 19.2% difference between the self-reported and the objective measures is comparable to the difference of approximately 17% estimated in a prior study [10]. However, another study among veterans did not find any significant differences between self-reported and objective (pill counts) measurements, wherein the mean objective adherence at 4-, 8-, and 12-week timepoints was 96.2%, 95.2%, and 98.2%, respectively, while the mean VAS adherence was 96.2%, 96.0%, and 98.2, respectively, at each of the timepoints [9]. This inconsistency between our study and the prior study by Burton et al. (2018) could be attributed to differences in the type of populations studied and to incorporation of an interviewer-assisted method for collecting self-reported data in the prior study. The accuracy of the self-reported data can be enhanced (i.e., the extent of overreporting can be reduced) through various measures such as including interviewer assistance [9], facilitating recall through use of optimal recall periods [28], measuring proportion rather than counts of medications taken, and reducing social desirability bias by letting respondent know that nonadherence is normal or avoiding face-to-face data collection [8]. However, our results may be applied only in contexts where such measures for enhancing self-reporting of adherence are not available or feasible. The comparability of our results to other studies may also be affected by the type of objective adherence measure used. A study reviewing different technology-based HCV DAA adherence measures found that the adherence ranges in studies using pill counts (> 98%) were higher compared to those using technology-based measures such as MEMS caps and ingestible sensors (95–97%), weekly adherence through electronic blister packs (73–98%), and electronic pill boxes (39–89%) [25].

Our study also examined how high overreporting of adherence relates to the treatment-related outcome of SVR. Overreporting adherence by ≥ 25% was determined as the optimal threshold for maximally predicting a lower SVR rate and was associated with ~ 9% lower SVR rate than those with < 25% overreported adherence. Within the self-reported adherence categories, the difference in SVR rates between < 25% vs. ≥ 25% overreporting was significant (*p* value < 0.05) or marginally significant (*p* value between 0.05 and 0.1) only for the higher ordered categories of self-reported adherence. An implication of this finding is that while those overreporting adherence at lower levels of self-reported adherence may still be recognized as having less than optimal adherence and receive support for improving adherence, low actual adherence may go unrecognized among those self-reporting high adherence, and they would likely be missed by interventions to support adherence.

Prior studies in the HIV prevention context found that age, educational level [13], and race [14] predicted overreporting of medication adherence. Our study found that characteristics such as unemployment and drug use characteristics were predictive of having overreporting ≥ 25%. Unemployment, a social determinant of health [29], is a personal resource barrier that reduces access to care and consequently affects HCV treatment compliance [30]. Substance use before or during treatment may interfere with HCV DAA adherence [11, 26]. Although clinical practices may not be able to identify overreporting patients at the individual level, knowledge about the factors/characteristics associated with overreporting may help identify patient populations at risk of high overreporting and support them for improving their actual adherence. Providing appropriate adherence supports that address the complex needs of the substance use population can help bridge the gap in adherence levels and SVR rates between people who use drugs and people who do not use drugs [31–33]. An example of an adherence support is the Toronto Community Hep C Program (TCHCP) which works collaboratively with community social support service agencies and features integration of a variety of services including primary care, infectious disease specialist, mental health, peer support, psycho-educational support groups, harm reduction program, case management, healthy meals, and travel support [32, 34]. In our study THC/cannabis use was associated with a protective effect against having ≥ 25% overreporting of adherence. There is evidence from the interferon era suggesting that cannabis use may facilitate HCV treatment adherence [35], an effect attributed to THC/cannabis ameliorating the severe adverse effects of interferon such as nausea [36]. These results support the existing advocacy for removal of cannabis use as a barrier for persons undergoing HCV treatment [36].

An added contribution of our study is examining the discrepancy between self-reported and objective adherence by varying levels of self-reported adherence. All the categories of self-reported adherence falling in the 80‒100% range had a substantially greater discrepancy between self-reported and objective adherence than the < 80% self-reported adherence category. No other studies have examined how overreporting varies based on the level of self-report. Because objective adherence measures are typically not available in clinical settings, the importance of our study is that our findings may help clinicians estimate patients’ actual level of adherence based on the self-reported adherence measure and provide additional support to help them succeed in maintaining adequate levels of adherence. Our results may also be used to gauge the level of bias introduced in treatment studies among PWID that rely on incorporating the exclusive use of the self-reported adherence measure.

Our study has some limitations. The study sample had relatively lower proportions of women, persons from minority racial/ethnic groups and persons from rural areas. The sample sizes for some categorical levels of self-reported adherence were small. Our study has several strengths. This is one of the first studies to focus on investigating how varying levels of discrepancy between self-reported and objective adherence measures relates to SVR. While overreporting adherence is common, we have determined a threshold of overreporting that is predictive of significantly worse chances of achieving SVR; the validity of this threshold for predicting SVR should be tested further in future studies. By exploring the correlates of problematic levels of overreported adherence with respect to achieving the SVR outcome, our study also helps define the subpopulation of PWID with HCV who may benefit from additional adherence support.

## Conclusions

Overreporting of adherence to HCV DAAs was greater at higher levels of self-reported adherence among PWID. Having ≥ 25% overreported adherence was associated with poorer chances of achieving HCV cure. Providers may need to intensively support PWID with risk factors for high overreporting to promote adherence and maximize the probability of SVR, possibly by addressing pressing needs such as finding employment and actively linking patients to substance use treatment.

## Data Availability

The dataset used for the current study is not publicly available because it contains information that could compromise the privacy of the research participants. The dataset is available from the corresponding author on reasonable request.

## Abbreviations

CDC: Centers for Disease Control and Prevention
CI: Confidence Interval
DAA: Direct-Acting Antiviral
GAD: Generalized Anxiety Disorder
HCV: Hepatitis C Virus
HERO: Hepatitis C Real Option
M: Mean
mDOT: Modified Directly Observed Therapy
MEMS: Medication Event Monitoring System
PHQ: Patient Health Questionnaire
PN: Patient Navigation
PWID: Persons who Inject Drugs
RNA: Ribonucleic Acid
ROC: Receiver Operating Characteristic
SD: Standard deviation
SVR: Sustained Virologic Response
THC: Tetrahydrocannabinol
VAS: Visual Analog Scale
